# The implication from RAS/RAF/ERK signaling pathway increased activation in epirubicin treated triple negative breast cancer

**DOI:** 10.18632/oncotarget.22604

**Published:** 2017-11-21

**Authors:** Jianbo Huang, Qingqing Luo, Yun Xiao, Hongyuan Li, Lingquan Kong, Guosheng Ren

**Affiliations:** ^1^ Department of Endocrine & Breast Surgery, The First Affiliated Hospital of Chongqing Medical University, Chongqing 400016, China; ^2^ Department of Oncology, Yongchuan Hospital of Chongqing Medical University, Chongqing 402160, China

**Keywords:** TNBC, RAS/RAF/ERK signaling pathway, GSEA, epirubicin, targeting therapy

## Abstract

**Background:**

Triple negative breast cancer (TNBC) is not sensitive to RAS/RAF/ERK signaling pathway (ERK pathway) targeting therapy, due to the absence of excessive activation of ERK pathway. However, the kinase cascades might be activated after chemotherapy in TNBC. Here we aimed to predict whether ERK pathway targeting therapy could be used as an adjuvant therapy in TNBC.

**Methods:**

Within online GEO datasets (GSE43816 and GSE54326), gene set enrichment analysis (GSEA) was performed to detect molecular changes in epirubicin treated TNBC samples and cells, ERK pathway components and regulation genes changes were included.

**Results:**

In epirubicin treated TNBC samples and cells, we found ERK pathway components (eg. MAPK13, MAP3K1, MAPK12, MAPK11 and MAPKAPK3) were obviously enriched, also, expression of ERK pathway positive regulation genes significantly increased (*P*<0.05) and negative regulation genes decreased (*P*<0.05) in epirubicin resistant cells. Moreover, phosphorylated ERK levels were significantly elevated in MDA-MB-231 cells after epirubicin treatment.

**Conclusion:**

ERK signaling pathway was more activated in epirubicin treated TNBC, possibly contributing to the epirubicin resistance in TNBC, it implicated that ERK pathway could be used as an novel candidate for targeting therapy in refractory and relapse TNBC.

## INTRODUCTION

ERK pathway is one of the best characterized kinase cascades in cancer cell biology [1]. It is triggered by either growth factors or activating mutations of major oncogenic proteins in this pathway, the most common being RAS and RAF. Deregulation of this pathway is frequently observed and plays a central role in several cancers, including melanoma, pancreatic, lung and colorectal cancer [2]. Targeting these kinases has already acquired a good effect in cancer therapy.

TNBC is defined as tumors that lack expression of estrogen receptor (ER), progesterone receptor (PR) and human epidermal growth factor receptor 2 (HER2), constitutes 10%-20% of all breast cancer, more frequently affects younger patient [3, 4]. TNBC tumors are generally larger in size, are of higher grade, have lymph node involvement at diagnosis, and are biologically more aggressive. It was reported that women with TNBC had shorter relapse free survival time than women with other types of breast cancer, less than 30% of women with metastatic TNBC survive 5 years, and almost all die of their disease despite adjuvant chemotherapy [5, 6]. Due to the less mutation frequency of ERK pathway kinases and lack expression of HER2, TNBC is poorly response to ERK pathway targeting therapy. Nevertheless, few studies concerned ERK pathway might be activated after chemotherapy in TNBC.

In this study, we investigated ERK pathway components and regulation genes changes after epirubicin treatment, tried to offer a novel targeting candidate for TNBC.

## RESULTS

### A retrospective overview of ERK pathway inhibitors in cancer treatment

We retrospectively reviewed recent publications on ERK pathway inhibitors for cancer treatment. As shown in Table 1, we could found ERK pathway inhibitors were widely applied in clinic, especially for patients harboring RAS or RAF mutations, which induced excessive and continuous activation of ERK pathway. Consequently, these patients always had a better prognosis after treatment of ERK pathway inhibitors. For breast cancer, it is less frequent to have RAS and RAF mutations compared with melanoma, colon cancer and non-small cell lung cancer (NSCLC), ERK pathway inhibitors were rarely used in breast cancer.

**Table 1:** Clinical trials evaluating RAS/RAF/ERK inhibitors, according to Clinical Trials.gov. (July, 2017)

Compound	Combination	Phase	Tumor	Results	Author, Year
Selumetinib (MEK1/2 inhibitor)	-	II	Myeloma (36)	The response rate (CR + PR) was 5.6% with a mean duration of response of 4.95 months and median progression-free survival time of 3.52 months. One patient had a very good partial response, 1 patient had a partial response, 17 patients had stable disease, 13 patients had progressive disease.	Holkova B, 2016
Vemurafenib (BRAF inhibitor)	-	II	Leukemia (54)	The overall response rates were 96% (25 of 26 patients who could be evaluated) after a median of 8 weeks in the Italian study and 100% (24 of 24) after a median of 12 weeks in the U.S. study. The rates of complete response were 35% (9 of 26 patients) and 42% (10 of 24) in the two trials, respectively.	Tiacci E, 2015
Selumetinib (MEK1/2 inhibitor)	dacarbazine	III	Uveal melanoma (estimated 128)	Ongoing	Carvajal RD, 2015
Trametinib (MEK1/2 inhibitor)	-	II	NSCLC (86)	10 (12%) patients had partial responses	Blumenschein GR Jr, 2015
Selumetinib (MEK1/2 inhibitor)	irinotecan	II	Colorectal (31)	3 patients (9.7 %) had partial response. 16 patients (51.6 %) had stable disease for ≥4 weeks, including three >1 year.	Hochster HS, 2015
Vemurafenib (BRAF inhibitor)		II	Papillary thyroid cancer (51)	Partial responses were recorded in ten of 26 patients in cohort 1.	Brose MS, 2016
Trametinib (MEK inhibitor)	Dabrafenib	II	Melanoma (23)	Disease control rate was 45%, and median PFS was 13 weeks.	Chen G, 2016
Selumetinib (MEK1/2 inhibitor)		II	Endometrial cancer (54)	Three (6%) patients had objective response (1 CR, 2 PR); 13 had SD as best response. The proportion of patients with 6-month EFS was 12%. Median EFS, progression-free and overall survival was 2.1, 2.3, and 8.5months, respectively.	Coleman RL, 2015
Trametinib (MEK inhibitor)	Dabrafenib	II	colorectal cancer (43)	Five (12%) achieved a partial response or better, including one (2%) complete response, with duration of response > 36 months; 24 patients (56%) achieved stable disease as best confirmed response.	Corcoran RB, 2015
Binimetinib (MEK inhibitor)		III	Melanoma (402)	Median progression-free survival was 2·8 months in the binimetinib group and 1·5 months in the dacarbazine group.	Dummer R, 2017
Selumetinib (MEK inhibitor)	temsirolimus	II	Soft-tissue sarcomas (71)	There was no difference in PFS between the two arms for the overall cohort; an improved median PFS was observed in the combination arm (N = 11) over single agent (N = 10) in the prespecified leiomyosarcoma stratum. Four-month PFS rate was 50% with the combination vs 0% with selumetinib alone in the leiomyosarcoma cohort.	Eroglu Z, 2015
Selumetinib (MEK1/2 inhibitor)	Erlotinib	II	Pancreatic Adenocarcinoma (46)	Although no objective responses were observed, 19 patients (41%) showed evidence of stable disease for ≥6 weeks, and 13 of 34 patients (38%) had a CA19-9 decline ≥50%. Median progression-free survival was 1.9 months, with a median overall survival of 7.3 months.	Ko AH, 2016
Vemurafenib (BRAF inhibitor)	II		Colorectal (21)	Of the 21 patients treated, one patient had a confirmed partial response and seven other patients had stable disease by RECIST criteria. Median progression- free survival was 2.1 months.	Kopetz S, 2015
Trametinib (MEK1/MEK2 inhibitor)		III	Melanoma (322)	The intent-to-treat (ITT) analysis estimated a 28% reduction in the hazard of death with trametinib treatment for patients in the primary efficacy population. Adjustment analyses deemed plausible provided OS HR point estimates ranging from 0.48 to 0.53. Similar reductions in the HR were estimated for the first-line metastatic subgroup. Treatment with trametinib, compared with chemotherapy, significantly reduced the risk of death and risk of disease progression in patients with BRAF V600E/K mutation-positive advanced melanoma or MM.	Latimer NR, 2016
Trametinib (MEK inhibitors) Dabrafenib (BRAF inhibitor)		II	NSCLC(57)	Thirty-six patients (63·2%) achieved an investigator-assessed overall response	Planchard D, 2016
Dabrafenib (BRAF inhibitor)		II	NSCLC(84)	Twenty-six of the 78 previously treated patients achieved an investigator-assessed overall response (33%). Four of the six previously untreated patients had an objective response.	Planchard D, 2016
Dabrafenib (BRAF inhibitor) and Trametinib (MEK inhibitor)		III	Melanoma(704)	The overall survival rate at 12 months was 72% in the combination-therapy group and 65% in the vemurafenib group. The prespecified interim stopping boundary was crossed, and the study was stopped for efficacy. Median progression-free survival was 11.4 months in the combination-therapy group and 7.3 months in the vemurafenib group. The objective response rate was 64% in the combination-therapy group and 51% in the vemurafenib group.	Robert C, 2015
Selumetinib (MEK 1/2 inhibitor)	Fulvestrant	II	Breast(46)	Recruitment was interrupted because the selumetinib-fulvestrant arm did not reach the pre-specified DCR. DCR was 23% in the selumetinib arm and 50%in the placebo arm. Median progression-free survival was 3.7months in the selumetinib arm and 5.6months in the placebo arm. Median time to treatment failure was 5.1 and 5.6 months, respectively.	Zaman K, 2015

### ERK pathway activation elevated in epirubicin treated TNBC samples

**Figure 1:**
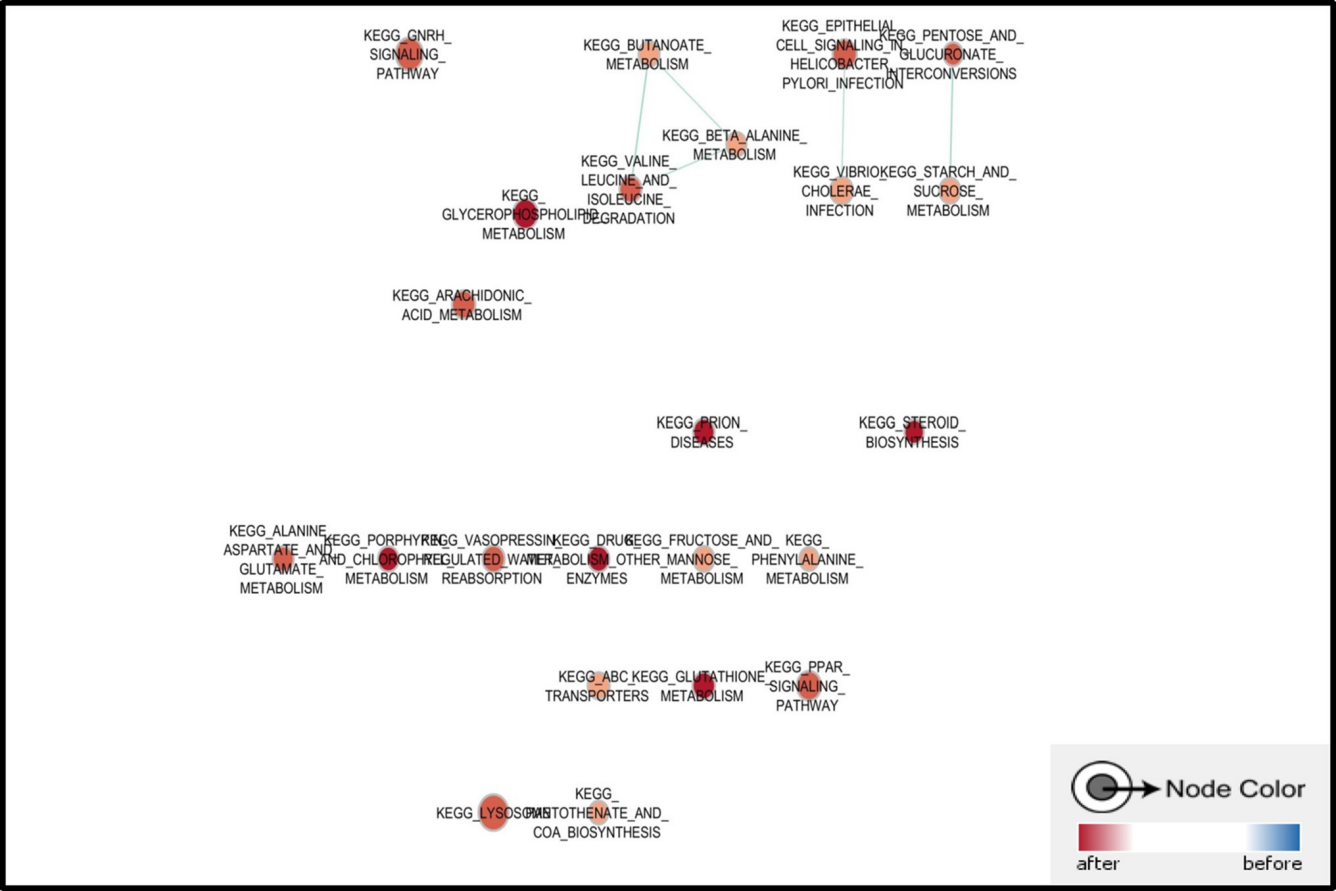
Pathways enriched in epirubicin treated TNBC sample Most of top enriched pathways were related with drug and molecule metabolism, also, ERK signaling pathway was enriched in epirubicin treated TNBC sample.

TNBC is always considered as a refractory subtype of breast cancer as survival for patients are shorter than other types of breast cancer, although some of them are well responsed to epirubicin contained chemotherapy, resistance and relapse still exist as the main challenge for TNBC treatment. Here we applied an online GEO dataset (GSE43816) for GSEA to discuss the molecular changes in epirubicin treated TNBC. This cohort study of 7 women with primary invasive TNBC were collected for tumor specimen before and after 4 cycles of NAC with epirubicine and cyclophosphamide, followed by 4 cycles of taxanes. Total RNA was extracted from tumor specimens and the whole transcriptome was quantified with Affymetrix HuGene 1.1 ST. Among top 20 enriched pathways in posttreatment tumors, most of them were related with drug and molecule metabolism. Additionally, ABC transporters related genes were also enriched in epirubicin treated samples, which supported these tumors were disposed to chemoresistance (Figure 1).

**Figure 2:**
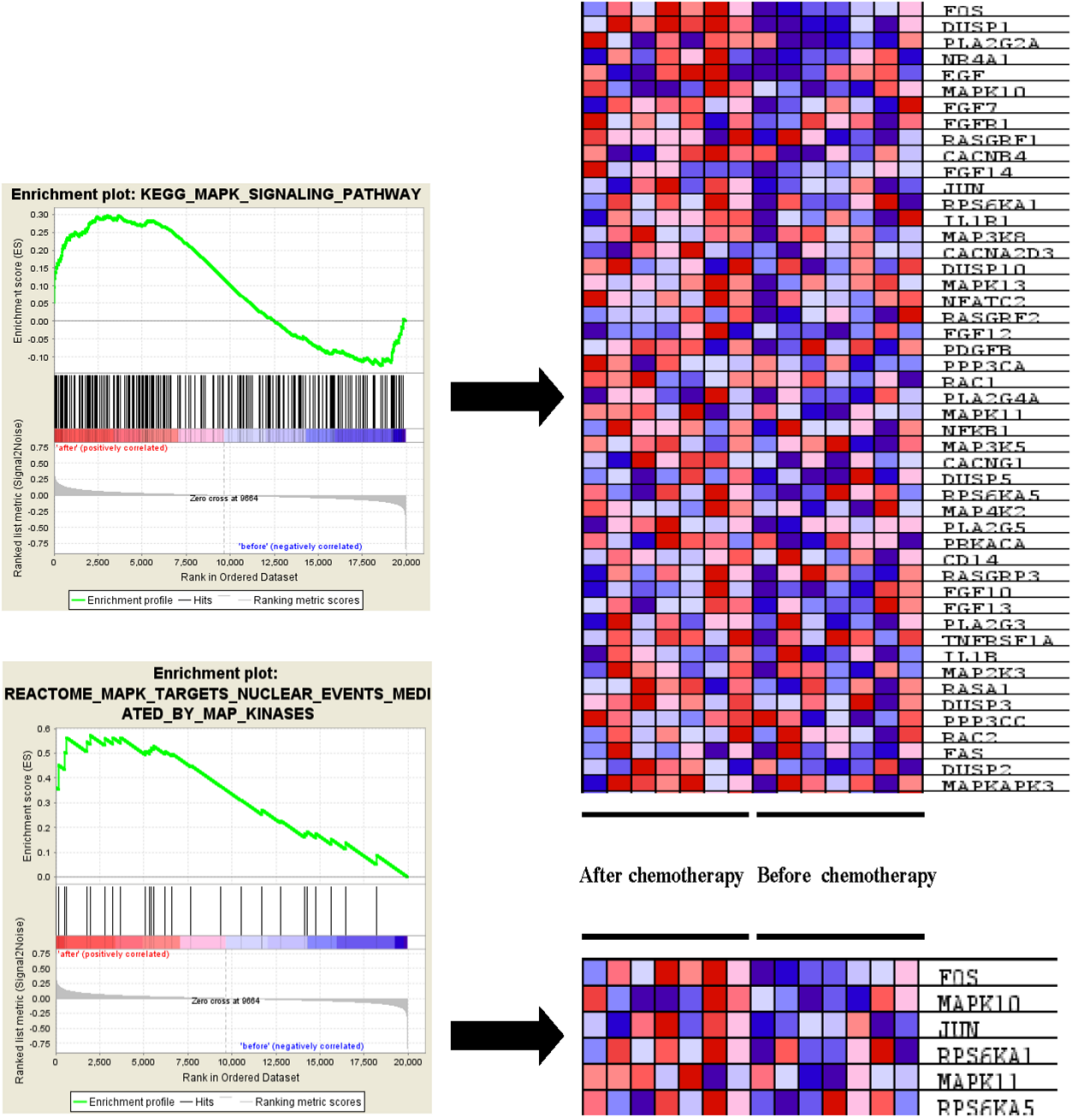
ERK pathway associated genes enriched in epirubicin treated TNBC sample ERK signaling pathway activators such as EGF, FGF7, FGF14 and FGFR1 were enriched in epirubicin treated TNBC sample.

It was also found that ERK pathway was enriched in tumor specimen after receiving chemotherapy, several ERK pathway component genes expression elevated in KEGG and REACTOME GSEA results, such as MAPK10, MAPK13, MAPK11 and MAPKAPK3 (Table 2). Also, pathway activators such as EGF, FGF7, FGF14 and FGFR1 were also enriched in epirubicin treated samples (Figure 2).

**Table 2:** MAPK family gene enriched in ERKsignaling pathway after neoadjuvant chemotherapy

Probe	Gene title	Rank metric score	Running ES	Core enrichment
*KEGG_ MAPK signaling pathway*				
MAPK10	mitogen-activated protein kinase 10	0.203	0.1633	Yes
MAPK13	mitogen-activated protein kinase 13	0.113	0.2426	Yes
MAPK11	mitogen-activated protein kinase 11	0.082	0.2517	Yes
MAPKAPK3	mitogen-activated protein kinase-activated protein kinase 3	0.056	0.2966	Yes
*REACTOME_MAPK_TARGETS_NUCLEAR_EVENTS_MEDIATED_BY_MAP_KINASES*				
MAPK10	mitogen-activated protein kinase 10	0.203	0.4514	Yes
MAPK11	mitogen-activated protein kinase 11	0.082	0.5460	Yes

### ERK pathway activation enhanced in epirubicin resistant TNBC cells

**Figure 3:**
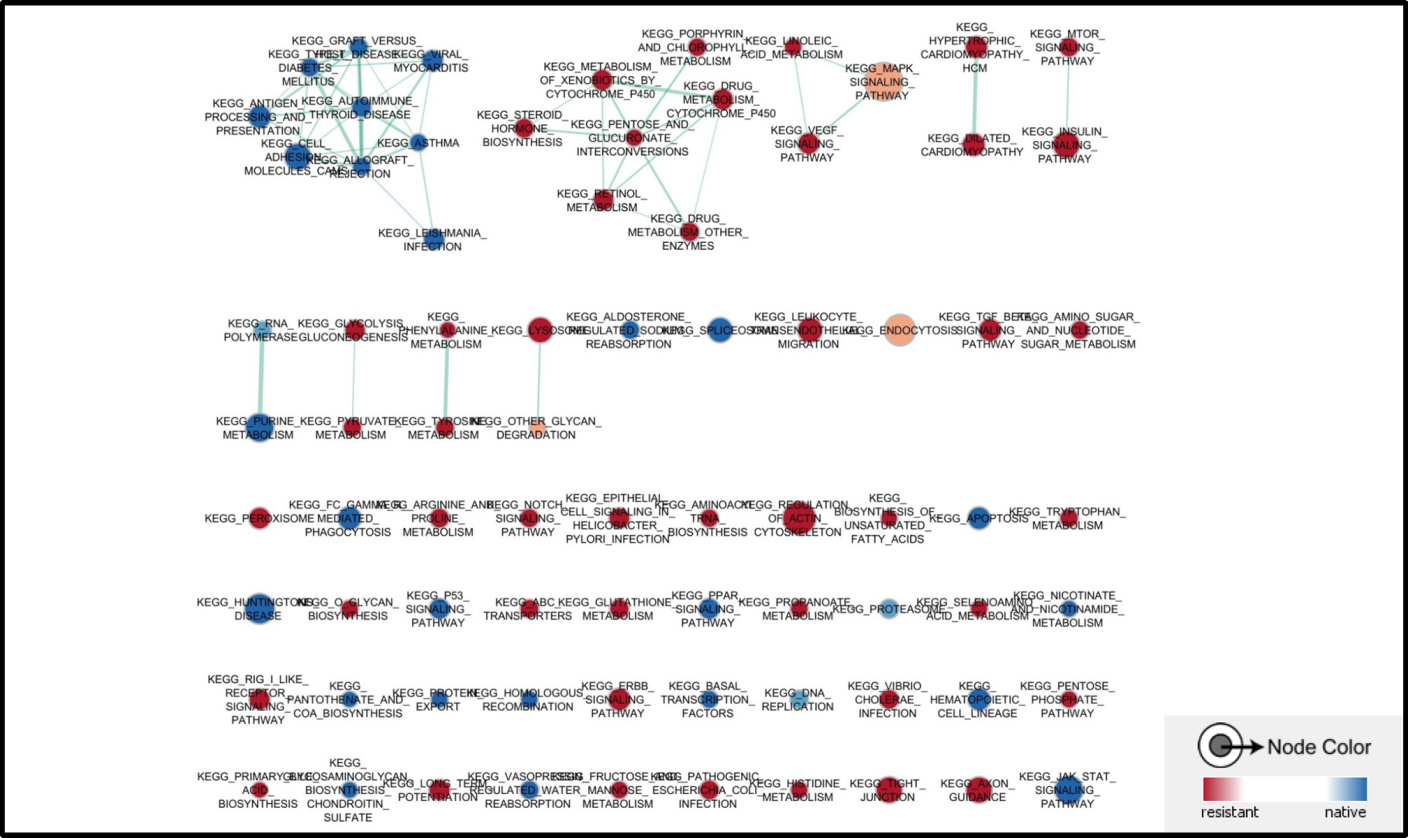
Pathways enriched in epirubicin resistant TNBC cells Drug and other molecules metabolism pathways were enriched in epirubicin resistant tumor cells. ABC transporters related genes expression was also enhanced in epirubicin resistant cells.

**Figure 4:**
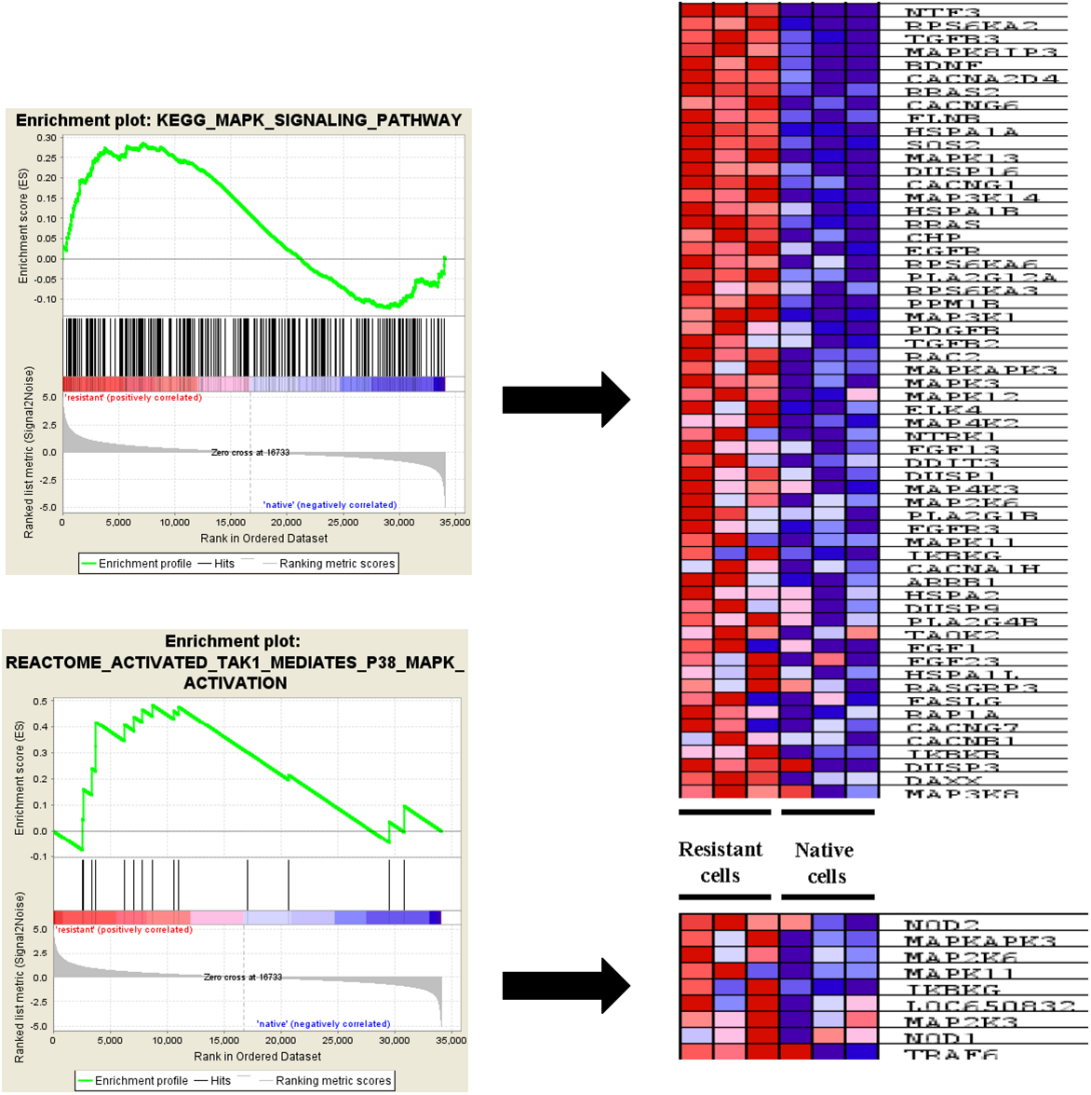
ERK pathway associated genes enriched in epirubicin resistant TNBC cells ERK signaling pathway component genes were enriched in epirubicin resistant cells, like MAPK8IP3, MAPK13 and MAP3K14. Other upstream genes of ERK signaling pathway such as EGFR, FGFR3 were also enriched in resistant tumor cells.

To avoid the heterogeneity of tumor sample from patients, here we applied a dataset (GSE54326) to determine the ERK pathway changes in epirubicin resistant TNBC cell line MDA-MB-231. Tumor cells were treated continuously within 25nM epirubicin to induce chemoresisrance. Epirubicin resistant cells were morphologically different from native cells, and had alterations in several signaling pathways, the whole transcriptome was quantified with illumina human HT-12 V4.0 expression beadchip. Similarly, it was also found that drug and other molecules metabolism pathways were enriched in epirubicin resistant tumor cells. ABC transporters related genes expression was also enhanced in epirubicin resistant cells (Figure 3). Additionally, we found more ERK signaling pathway component genes were enriched in epirubicin resistant cells, like MAPK8IP3, MAPK13 and MAP3K14, etc. (Table 3). Other upstream genes of ERK signaling pathway such as EGFR, FGFR3 were also enriched in resistant tumor cells (Figure 4).

**Table 3:** MAPK family gene enriched in ERK signaling pathway in epirubicin resistant MDA-MB-231 cells

Probe	Gene title	Rank metric score	Running ES	Core enrichment
KEGG_ MAPK signaling pathway				
MAPK8IP3	mitogen-activated protein kinase 8 interacting protein 3	2.315	0.0635	Yes
MAPK13	mitogen-activated protein kinase 13	1.662	0.1400	Yes
MAP3K14	mitogen-activated protein kinase kinasekinase 14	1.502	0.1608	Yes
MAP3K1	mitogen-activated protein kinase kinasekinase 1	1.151	0.2085	Yes
MAPKAPK3	mitogen-activated protein kinase-activated protein kinase 3	1.055	0.2247	Yes
MAPK3	mitogen-activated protein kinase 3	1.039	0.2289	Yes
MAPK12	mitogen-activated protein kinase 12	1.038	0.2352	Yes
MAP4K2	mitogen-activated protein kinase kinasekinasekinase 2	1.006	0.2440	Yes
MAP4K3	mitogen-activated protein kinase kinasekinasekinase 3	0.916	0.2595	Yes
MAP2K6	mitogen-activated protein kinase kinase 6	0.909	0.2636	Yes
MAPK11	mitogen-activated protein kinase 11	0.850	0.2689	Yes
MAP3K8	mitogen-activated protein kinase kinasekinase 8	0.569	0.2737	Yes
MAP2K3	mitogen-activated protein kinase kinase 3	0.499	0.2806	Yes
REACTOME_ACTIVATED_TAK1_MEDIATES_P38_MAPK_ACTIVATION				
MAPKAPK3	mitogen-activated protein kinase-activated protein kinase 3	1.055	0.1597	Yes
MAP2K6	mitogen-activated protein kinase kinase 6	0.909	0.2390	Yes
MAPK11	mitogen-activated protein kinase 11	0.850	0.3228	Yes
MAP2K3	mitogen-activated protein kinase kinase 3	0.499	0.4379	Yes

Moreover, we selected top 5 MAPK family genes to run a real time PCR, and found mRNA expression change is consistent with the GSEA analysis results, all *P* value is less than 0.05 (Supplementary Figure 1A).

### ERK pathway positive and negative regulation genes expression in epirubicin resistant TNBC cells

**Figure 5:**
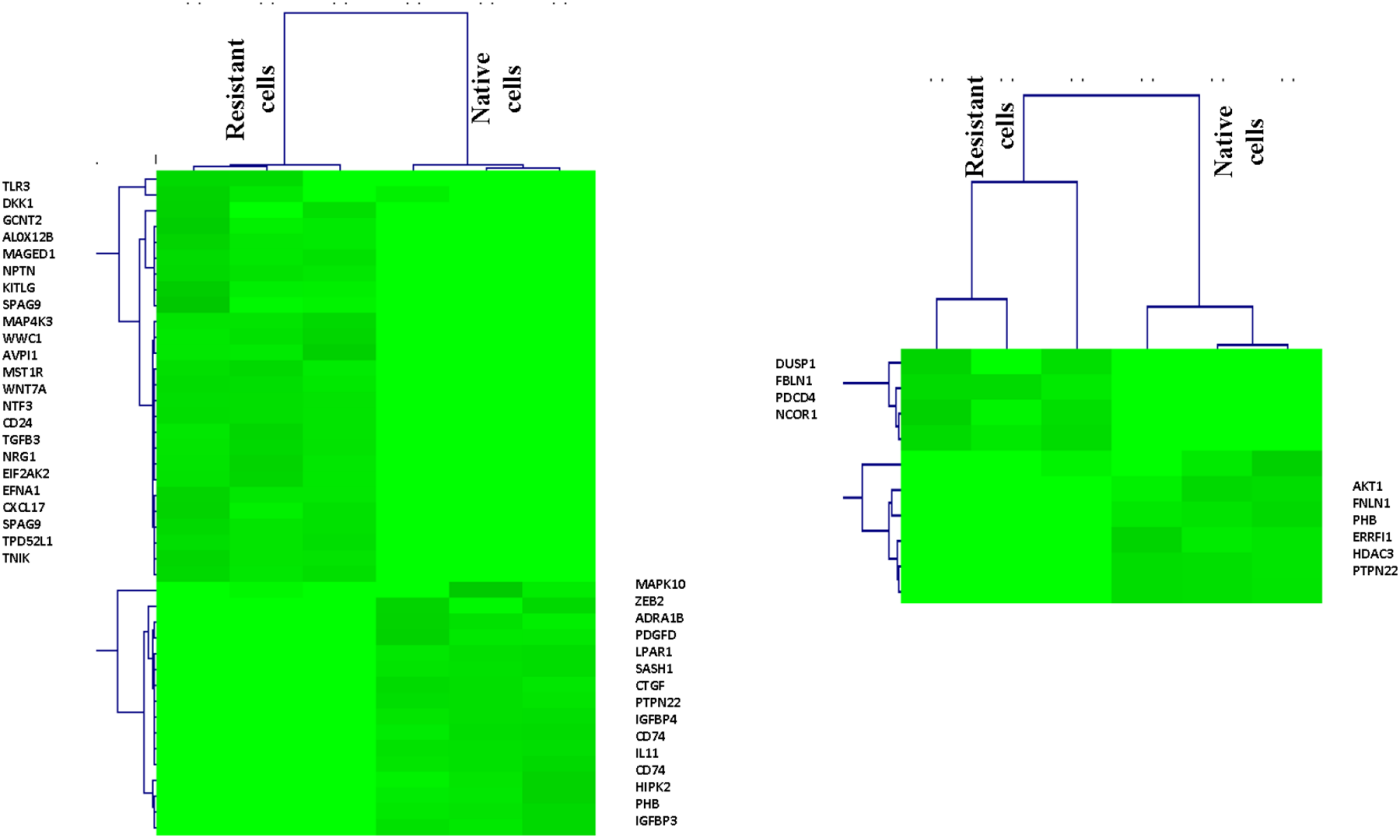
Positive and negative regulation genes of ERK pathway in TNBC cells Most positive regulation genes were enriched in epirubicin resistant tumor cells and most negative regulation genes were enriched in native tumor cells.

To better understand ERK pathway change after epirubicin treatment, we investigated the positive and negative regulation genes, the gene lists were downloaded from AmiGO2 (http://geneontology.org/) and shown in Supplementary Tables 1 and 2. Consequently, as shown in the heat map, we found most positive regulation genes were enriched in epirubicin resistant tumor cells (*P*< 0.05) and most negative regulation genes were enriched in native tumor cells (*P*< 0.05) (Figure 5). This finding further proved that ERK signaling pathway could be activated in epirubicin resistant tumor cells. Moreover, we used western blot to determine ERK phosphorylation changes in MDA-MB-231 after epirubicin treatment. Similarly, we found ERK phosphorylation level was elevated after 5μM epirubicin treatment (Supplementary Figure 1B).

## DISCUSSION

The ERK pathway is one of the best-characterized kinase cascades in cancer cell biology. It is triggered by growth factors or activating mutations of oncogenic kinases such as KRAS, BRAF involved in this pathway. Deregulation of the ERK pathway is observed in several cancers and yields multiple changes in the expression of numerous genes involved in tumor cell differentiation, proliferation, survival, migration, and angiogenesis [1]. Kinases of this pathway are promising targets for identifying novel therapies.

Presently, mounts of inhibitors to ERK signaling pathway are being tested in clinical trials, some of them have already been successfully applied in cancer treatment and emerge as an optional therapy for patients with refractory and relapsed tumor [7–10]. However, most of tumors responsing to the targeting therapy possess oncogenic kinases mutations, the antitumor activity always relies on degrade of activation of ERK pathway [11, 12]. Within retrospective review of recent studies about ERK pathway inhibitors, we found melanoma, NSCLC and colon cancer were always well responsed to targeting therapy [11, 13, 14]. It was reported that breast cancer rarely possessed ERK pathway kinases mutations, BRAF (2%), KRAS (5%), and HRAS (1%) mutations occur at even lower frequency in TNBC [15, 16], only a few of studies recognized breast cancer as a good candidate of ERK pathway targeting therapy. TNBC were significantly more sensitive to MEK inhibition relative to luminal and HER2 amplified lines [16], due to having an activated RAS like transcriptional program [15].

Chemotherapy remains the important strategy for TNBC treatment following mastectomy, it has elevated a lot in 5 years overall survival rate since it becomes the standard adjuvant therapy for breast cancer. Epirubicin, taxol and cyclophosphamide compose the first line of chemotherapeutics recommended by the guideline [17]. Nevertheless, drug resistance in TNBC is the major obstacle to a successful outcome following chemotherapy treatment. While up-regulation of multidrug resistance (MDR) genes is a key component of drug resistance in multiple cancers, the complexity and hierarchy of non-MDR driven drug resistance pathways are still largely unknown [18]. There are studies identifying pathways and genes contributing to drug resistance, which contribute to several biological pathways, including cell cycle, chromosomal maintenance, epigenetics, RNA and mitochondrial transcription [19–21]. In our study, we applied two GEO online datasets of genes expression to analyze the molecule changes after chemotherapeutics, especially for ERK pathway change.

In our GSEA results, we found most pathways enriched in chemotherapeutics treated tumor samples and cells were associated with drug metabolism and ABC transporter, which was consistent with the previous findings about chemoresistance. Also, ERK pathway was enriched in tumor samples receiving chemotherapy, which contributed to the resistance and relapse in TNBC. In epirubicin resistant TNBC cells, ERK pathway enrichment was further proved within GSEA and enrichment map analysis. This interesting finding implicated that epirubicin resistant subtype of TNBC could be an optimal candidate for ERK pathway targeting therapy. To make a further validation, we explored and found ERK phosphorylation elevation in MDA-MB-231 cells treated with epirubicin, which further implicated ERK pathway inhibitors could be used together with epirubicin or in epirubicin resistant cells.

In summary, we firstly proved that chemotherapeutics especially epirubicin could enhance ERK phosphorylation in TNBC. The hints of ERK pathway inhibitors application in TNBC after receiving chemotherapyshould be promising but needs a proof in clinic.

## MATERIALS AND METHODS

### GEO datasets, GSEA, enrichment map analysis and heatmap

GEO datasets GSE43816 and GSE54326 were downloaded from website (https://www.ncbi.nlm.nih.gov/), GSEA, enrichment map analysis and heat map were performed with R software.

### Materials and cell cultures

The monoclonal antibody against GAPDH was purchased from Epitomics (Burlingame, CA). The p-ERK, ERK antibodies were purchased from Cell Signaling Technology (Danvers, MA). The human breast cancer cell line MDA-MB-231 used was kindly provided by the Chinese University of Hong Kong. The cancer cells were maintained in RPMI 1640 (Gibco-BRL, Karlsruhe, Germany) supplemented with 10% fetal bovine serum (PAA Laboratories, Linz, Austria), 100 U/ml penicillin and 100 mg/ml streptomycin and were cultured at 37°C in a humidified atmosphere containing 5% CO_2_.

### Protein preparation

Protein was isolated as literatures described. Briefly, protein was extracted using RIPA buffer (Beyotime, Shanghai, China) containing a mixture of protease inhibitors and phosphatase inhibitors (Sigma-Aldrich, Saint Louis, MI). After extraction, the protein was quantified using a BCA kit (Thermo Fisher Scientific). The protein samples were denatured then stored at -20°C until use.

### Western blot analysis

Western blot analysis was performed as literature described. Briefly, 40 μg of protein from each sample was separated by SDS-PAGE according to molecular weight. The proteins were transferred to an equilibrated polyvinylidene difluoride membrane (Amersham Biosciences, Buckinghamshire, UK) and then incubated with a specific primary antibody at 4°C overnight. After incubation with the secondary antibody, the proteins were detected by enhanced chemiluminescence. The band intensity was quantified with Quantity One software.

### Statistics

Statistical analysis was done using Student's t-test, variance analysis and/or non-parametric tests. For all the tests, *P*<0.05 was considered as statistical significance. All statistics were calculated by SPSS 13.0 or R software.

## SUPPLEMENTARY MATERIALS FIGURES AND TABLES





## Abbreviations

TNBC: triple negative breast cancer
GSEA: gene set enrichment analysis
ER: estrogen receptor
PR: progesterone receptor
HER2: epidermal growth factor receptor 2
NSCLC: non-small cell lung cancer
MDR: multidrug resistance.
