# Supplementary material for: The implication from RAS/RAF/ERK signaling pathway increased activation in epirubicin treated triple negative breast cancer
Source: Oncotarget. 2017 Nov 21;8(64):108249–60. doi: 10.18632/oncotarget.22604 (PMC5746140; doi:10.18632/oncotarget.22604)
Supplement: Supplementary file 1 [file oncotarget-08-108249-s001.pdf]

The implication from RAS/RAF/ERK signaling pathway increased activation in epirubicin treated triple negative breast cancer

SUPPLEMENTARY MATERIALS

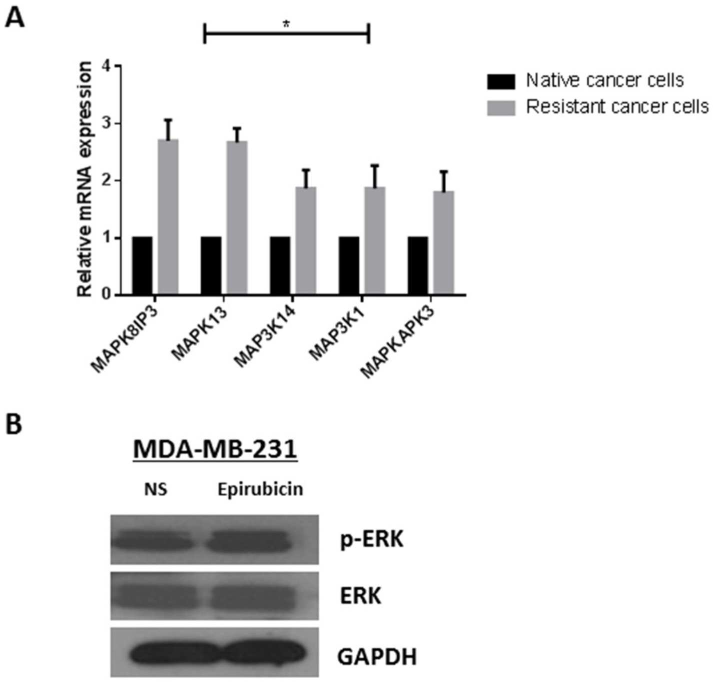

* *P*<0.05

Supplementary Figure 1: ERK signaling pathway activation after epirubicin treatment. **(A)** MAPK family genes mRNA expression increased in epirubicin resistant cells. **(B)** ERK phosphorylation level was elevated after 5μM epirubicin treatment.

Supplementary Table 1: Gene list of positive regulation of ERK signaling pathway

See Supplementary File 1

Supplementary Table 2: Gene list of negative regulation of ERK signaling pathway

ABL1	ERRFI1	NLRP6	SPRY2
ADIPOQ	EZR	NUP62	SPRY4
AIDA	F2RL1	P2RX7	STK38
AKT1	FBLN1	PAFAH1B1	SYNJ2BP
AMBP	FKTN	PAQR3	TAOK3
APOE	FLCN	PBK	TBC1D10C
ARRB1	FOXM1	PDCD4	TIMP3
ASH1L	FOXO1	PER1	TLR4
ATF3	GBA	PHB	TNIP1
BMP4	GBP1	PIN1	TP73
BMP7	GPER1	PINK1	UCHL1
C1QL4	GPS1	PPEF2	VRK3
C3orf33	GPS2	PPP2CA	WNK2
CAV1	GSTP1	PPP2R1A	ZMYND11
CAV3	HDAC3	PRKCD	ZNF675
CBLC	HIPK3	PRKN	SPRY2
CD300A	HMGCR	PSMD10	SPRY4
CDK5RAP3	HSPH1	PTEN	STK38
CNKSR3	HYAL2	PTPN1	SYNJ2BP
CRYBA1	IGBP1	PTPN2	TAOK3
CSK	IGF1R	PTPN22	TBC1D10C
DAB2IP	IL1B	PTPN6	TIMP3
DACT1	INPP5K	PTPRJ	TLR4
DAG1	IRAK3	PTPRR	TNIP1
DLG1	ITCH	QARS	TP73
DNAJA1	ITGB1BP1	RANBP9	UCHL1
DUSP1	KLF4	RAPGEF1	VRK3
DUSP10	LAX1	RGS14	WNK2
DUSP14	LEMD2	RGS2	ZMYND11
DUSP16	LIF	RGS3	ZNF675
DUSP18	LMO3	RGS4	SPRY2
DUSP19	LYN	RNF149	SPRY4
DUSP2	MAPK7	RPS6KA6	STK38
DUSP21	MAPK8IP1	SERPINB3	SYNJ2BP
DUSP22	MARVELD3	SFRP1	TAOK3
DUSP26	MBIP	SFRP2	TBC1D10C
DUSP3	MECOM	SIRT3	TIMP3
DUSP4	MEN1	SLC9A3R1	TLR4
DUSP5	MYC	SMAD4	TNIP1
DUSP6	NCOR1	SMPD1	TP73
DUSP7	NDRG2	SORL1	UCHL1
DUSP8	NF1	SPRED1	VRK3
DUSP9	NF2	SPRED2	WNK2
EIF3A	NLRP12	SPRY1	ZMYND11
