# Supplementary material for: The implication from RAS/RAF/ERK signaling pathway increased activation in epirubicin treated triple negative breast cancer
Source: Oncotarget. 2017 Nov 21;8(64):108249–60. doi: 10.18632/oncotarget.22604 (PMC5746140; doi:10.18632/oncotarget.22604)
Supplement: Supplementary file 2 [file oncotarget-08-108249-s002.docx]

| ABCA7 | C5AR1 | CDON  **Table S1. Gene list of positive regulation of ERK signaling pathway** | EPHA8 | FZD8 | IL26 |
| --- | --- | --- | --- | --- | --- |
| ABL1 | C5AR2 | CFLAR | EPO | GADD45A | IL6 |
| ACKR3 | CARD9 | CHI3L1 | ERBB2 | GADD45B | ILK |
| ADAM8 | CARTPT | CHRNA7 | ERBB4 | GADD45G | INS |
| ADAM9 | CAV2 | CIB1 | ERCC6 | GAREM1 | INSR |
| ADCYAP1 | CCL1 | CRK | ERN1 | GAS6 | IQGAP1 |
| ADORA1 | CCL11 | CRKL | ERN2 | GCG | IQGAP3 |
| ADORA2B | CCL13 | CSF1R | ERP29 | GCNT2 | IRAK1 |
| ADRA1A | CCL14 | CSK | EZH2 | GDF6 | IRAK2 |
| ADRA1B | CCL15 | CSPG4 | F2R | GH1 | ITGA1 |
| ADRA2A | CCL16 | CTGF | F2RL1 | GHR | ITGAV |
| ADRA2B | CCL17 | CTNNB1 | FAM58A | GHRL | JAK2 |
| ADRA2C | CCL18 | CX3CL1 | FBXW7 | GLIPR2 | JUN |
| ADRB2 | CCL19 | CXCL17 | FCER1A | GNAI2 | KARS |
| ADRB3 | CCL2 | CXCR4 | FFAR4 | GNG3 | KDR |
| AGER | CCL20 | CYSLTR2 | FGA | GPER1 | KIDINS220 |
| AJUBA | CCL21 | DAB2IP | FGB | GPNMB | KISS1 |
| AKAP13 | CCL22 | DAXX | FGD2 | GPR183 | KIT |
| ALK | CCL23 | DBNL | FGF1 | GPR37 | KITLG |
| ALKAL1 | CCL24 | DKK1 | FGF10 | GPR37L1 | KL |
| ALKAL2 | CCL25 | DNAJC27 | FGF18 | GPR55 | KLB |
| ALOX12B | CCL26 | DRD2 | FGF19 | GRM1 | KRAS |
| ALOX15 | CCL3 | DRD4 | FGF2 | GRM4 | KSR1 |
| ANGPT1 | CCL3L1 | DSTYK | FGF20 | HACD3 | KSR2 |
| ANKRD6 | CCL4 | DUSP15 | FGF21 | HAND2 | LAMTOR1 |
| AR | CCL4L1 | DUSP19 | FGF23 | HAVCR2 | LAMTOR2 |
| ARAF | CCL5 | DUSP22 | FGF4 | HCRTR1 | LAMTOR3 |
| ARHGAP8 | CCL7 | DUSP5 | FGF8 | HGF | LEP |
| ARHGEF5 | CCL8 | DUSP6 | FGF9 | HIPK2 | LGALS9 |
| ARL6IP5 | CCR1 | DUSP7 | FGFR1 | HMGB1 | LIF |
| ARRB1 | CCR7 | DUSP9 | FGFR2 | HMGCR | LPAR1 |
| ARRB2 | CD24 | DVL2 | FGFR3 | HRAS | LPAR3 |
| ATP6AP1 | CD27 | DVL3 | FGFR4 | HTR2A | LRRK2 |
| AVPI1 | CD36 | EDA2R | FGG | HTR2B | LTBR |
| AXIN1 | CD4 | EDAR | FLT1 | HTR2C | MADD |
| BANK1 | CD40 | EDN1 | FLT3 | ICAM1 | MAGED1 |
| BIRC7 | CD40LG | EDN3 | FLT4 | IGF1 | MAP2K1 |
| BMP2 | CD44 | EFNA1 | FPR1 | IGF1R | MAP2K2 |
| BMP4 | CD74 | EGF | FRS2 | IGF2 | MAP2K3 |
| BMPER | CD81 | EGFR | FSHR | IGFBP3 | MAP2K4 |
| BRAF | CDC42 | EIF2AK2 | FZD10 | IGFBP4 | MAP2K5 |
| C1QTNF1 | CDH2 | ELANE | FZD4 | IKBKG | MAP2K6 |
| C1QTNF2 | CDK1 | EPGN | FZD5 | IL11 | MAP2K7 |
| C5 | CDK10 | EPHA4 | FZD7 | IL1B | MAP3K1 |
| MAP3K10 | NEK10 | PIK3R5 | SASH1 | TLR9 |  |
| MAP3K11 | NELFE | PIK3R6 | SCIMP | TNF |  |
| MAP3K12 | NENF | PKN1 | SDCBP | TNFAIP8L3 |  |
| MAP3K13 | NGF | PLA2G1B | SEMA4C | TNFRSF11A |  |
| MAP3K14 | NOD1 | PLA2G2A | SEMA7A | TNFRSF19 |  |
| MAP3K15 | NOD2 | PLA2G5 | SERPINF2 | TNFSF11 |  |
| MAP3K2 | NODAL | PLCB1 | SHC1 | TNIK |  |
| MAP3K20 | NOX1 | PLCE1 | SHC2 | TP73 |  |
| MAP3K21 | NOX4 | PLCG1 | SLAMF1 | TPD52L1 |  |
| MAP3K3 | NPNT | PRKAA1 | SLC30A10 | TRAF2 |  |
| MAP3K4 | NPTN | PRKCA | SOD1 | TRAF4 |  |
| MAP3K5 | NPY5R | PRKCDBP | SORBS3 | TRAF6 |  |
| MAP3K6 | NQO2 | PRKCE | SOX2 | TRAF7 |  |
| MAP3K7 | NRG1 | PRKCZ | SPAG9 | TREM2 |  |
| MAP3K7CL | NRK | PRKD2 | SPRY2 | TRIM5 |  |
| MAP3K8 | NRP1 | PRMT1 | SRC | TRPV4 |  |
| MAP3K9 | NTF3 | PROK1 | SSTR4 | UBA52 |  |
| MAP4K1 | NTRK1 | PROK2 | STK25 | UBB |  |
| MAP4K2 | NTRK2 | PSAP | STK3 | UBC |  |
| MAP4K3 | NTRK3 | PSEN1 | SYK | UBE2N |  |
| MAP4K5 | OPRK1 | PTEN | SYT14P1 | UBE2V1 |  |
| MAPK1 | OPRM1 | PTK2B | TAB1 | UNC5CL |  |
| MAPK10 | OSM | PTPN1 | TAB2 | VANGL2 |  |
| MAPK11 | P2RX7 | PTPN11 | TAB3 | VEGFA |  |
| MAPK14 | P2RY1 | PTPN22 | TAOK1 | WNT16 |  |
| MAPK3 | PAK1 | PYCARD | TAOK2 | WNT5A |  |
| MAPK8IP2 | PAK3 | RAF1 | TAOK3 | WNT7A |  |
| MAPK8IP3 | PDCD10 | RAP1A | TBX1 | WNT7B |  |
| MAPKAPK2 | PDE5A | RAPGEF1 | TDGF1 | WWC1 |  |
| MAPKAPK3 | PDE6G | RAPGEF2 | TEK | XCL1 |  |
| MAPKAPK5 | PDE6H | RASGRP1 | TENM1 | XCL2 |  |
| MDFIC | PDE8A | RASSF2 | TGFA | XDH |  |
| MID1 | PDGFA | RB1CC1 | TGFB1 | ZC3H12A |  |
| MIF | PDGFB | RIPK1 | TGFB2 | ZEB2 |  |
| MINK1 | PDGFC | RIPK2 | TGFB3 | ZNF622 |  |
| MOS | PDGFD | RIT2 | TGFBR1 |  |  |
| MST1R | PDGFRA | ROR2 | THBS1 |  |  |
| MT3 | PDGFRB | RPS27A | THPO |  |  |
| MUC20 | PEA15 | RPS3 | TIAM1 |  |  |
| MUL1 | PELI2 | RYK | TIMP2 |  |  |
| MYDGF | PHB | S100A12 | TIRAP |  |  |
| NCF1 | PHB2 | S100A7 | TLR3 |  |  |
| NDRG4 | PIK3CB | S1PR2 | TLR4 |  |  |
| NECAB2 | PIK3CG | SAA1 | TLR6 |  |  |
